# Early mechanisms of neutrophil activation and transmigration in acute lung injury

**DOI:** 10.3389/fphys.2022.1059686

**Published:** 2022-12-21

**Authors:** L. A. Cagle, A. L. Linderholm, L. M. Franzi, J. A. Last, S. I. Simon, N. J. Kenyon, R. W. Harper

**Affiliations:** ^1^ University of California Lung Center, University of California, Davis, Davis, CA, United States; ^2^ Division of Pulmonary, Critical Care and Sleep Medicine, University of California, Sacramento, Sacramento, CA, United States; ^3^ Department of Veterans Affairs, Northern California Healthcare System, Mather, CA, United States; ^4^ Department of Biomedical Engineering, University of California, Davis, Davis, CA, United States

**Keywords:** neutrophil migration, dual oxidase, neutrophil activation, acute lung injury, mouse model, CD18, CD18-independent, LPS

## Abstract

**Introduction:**Neutrophil transmigration is multifactorial and primarily driven by selectins and β_2_-integrins (CD11b/CD18), whose expression are dependent on the underlying stimulus. Ventilator-induced lung injury (VILI) results in a predominantly CD18-independent mechanism of neutrophil recruitment, while direct endotoxin-induced lung injury results from a CD18-dependent mechanism. We previously observed that lack of NADPH oxidases DUOX1 and DUOX2 resulted in reduced neutrophil influx in a VILI model of lung injury but had no influence on neutrophil influx after LPS exposure. Based on these observations, we hypothesized that DUOX1/DUOX2 are an important component of CD18-independent mechanisms of neutrophil recruitment in the lung.

**Methods:**We exposed *Duoxa* ^*−/−*^ (KO) mice and *Duoxa* ^*+/+*^ (WT) mice to either an intratracheal exposure of lipopolysaccharide (LPS/endotoxin)-or high tidal volume ventilation and compared expression of neutrophil markers between groups. WT mice (129S6/SvEvTac) were obtained from Taconic Biosciences (One Discovery Drive Suite 304; Rensselaer, NY 1244) and were allowed to acclimatize for one week prior to study enrollment. KO mice were generated as previously described [Grasberger 2012] and bred in-house on a 129S6 background. We provided positive-pressure ventilation at a tidal volume of 10 ml/kg with 2 cmH20 positive end-expiratory pressure (PEEP). Mice were assigned to groups consisting of KO (n = 5) and WT (n = 5) in each group and divided into non-ventilated, positive-pressure ventilation, or LPS IT exposure groups. Positive-pressure ventilation was instituted for 4-h using a FlexiVent (Flexiware 8.1, Scireq, Montreal, QC, Canada). Lipopolysaccharide (Salmonella enterica serotype tryphimurium L6143, Millipore Sigma) was administered via an intratracheal (IT) route at a dose of 0.1 mg/kg. Mice were humanely euthanized at 4-h post-injection consistent with the UC Davis IAUCAC-approved protocol.

**Results:**As previously observed, neutrophilic influx into the airways was significantly impaired in the *Duoxa* ^*−/−*^ (KO) mice after VILI, but not after LPS exposure. LPS-induced lung injury resulted in upregulation of CD11b^+^ neutrophils and shedding of CD62L and CD162 regardless of DUOX expression, whereas VILI resulted in upregulation of CD49^+^ neutrophils in the *Duoxa* ^*+/+*^ (WT) mice but not the *Duoxa* ^*−/−*^ (KO) mice.

**Conclusion:**Our data suggest DUOX is required for CD18-independent mechanisms of neutrophil recruitment in the lung induced by acute lung injury, but not for canonical CD18depedent mechanisms after LPS exposure.

## Introduction

Neutrophil migration in the lung is a complex interplay between activated neutrophils, pulmonary endothelium, basement membrane and alveolar epithelium (Burns et al., 2003; Zemans et al., 2009). Adding to this complexity, the mechanisms of neutrophil migration in the lung varies according to the stimulus. One major differentiating factor is the utilization of CD11b/CD18. LPS-induced peritonitis in mouse models of endotoxemia and sepsis result in neutrophil migration by down-regulation of CD62L (L-selectin) and upregulation of CD11b/CD18 on the neutrophil cell surface (Chakrabortyet al, 2015; Soler-Rodriguez et al., 2000). In contrast, ventilator-induced lung injury (VILI) results in alveolar neutrophil migration independent of CD18 integrin-mediated interactions (Choudhury et al., 2004). Ridger et al. (2001) previously identified that CD49d mediated CD18-independent neutrophil migration in pulmonary inflammation. They determined that CD49d was involved in KC (CXC chemokine) but not LPS-induced responses, suggesting a CD18-independent neutrophil migration (Ridger et al., 2001). Some bacteria induce CD18-dependent neutrophil influx, whereas intraperitoneal injection of LPS or ventilator-induced lung injury (VILI) results in CD18-independent neutrophil influx (Reuthershan and Ley, 2004). There is currently a paucity of *in vivo* models that explore potential mechanisms responsible for these differences.

We previously demonstrated that dual NADPH oxidases, DUOX1/DUOX2, expressed in ciliated cells in the airways and type II cells of the alveoli (Fischer 2009), were an essential factor for neutrophil recruitment into the alveolar space by utilizing an ovalbumin-induced asthma model (Chang et al., 2013). In a follow-up study, we further evaluated the role of DUOX1/DUOX2 in neutrophil migration using a VILI model and LPS-induced lung injury model. We discovered that the presence of dual oxidases was essential for neutrophil migration only in our VILI model (Cagle et al., 2021). These differences suggested to us that, in contrast to LPS-induced neutrophil sequestration in the lung, dual oxidases may be an essential component of CD18-independent neutrophil recruitment during VILI. We specifically focused on expression of very late antigen 4 (CD49d) as a marker of CD18-independent mechanisms (Tasaka et al., 2002). Given our previous observations, we hypothesized that CD49d was involved in ventilator-induced lung injury but not LPS-induced neutrophil influx.

We sought to further understand the role of DUOX in regulating CD18- independent mechanisms of neutrophil recruitment by comparing changes in neutrophil marker expression in *Duoxa* ^*−/−*^ (KO) mice and *Duoxa* ^*+/+*^ (WT) mice after LPS exposure or VILI. The dual oxidase maturation factors (DUOXA1 and DUOXA2) form heterodimeric complexes with their respective DUOX1 and DUOX2 enzymes and are required for full functionality for their respective protein. In the *Duoxa* ^*−/−*^ mice, used in this study, both *DUOXA1* and *DUOXA2* genes are deleted, resulting in loss of DUOX1 and DUOX2 functional activity (Grasberger et al., 2012). This allowed us to specifically study the role of DUOX plays in CD18-independent neutrophil recruitment.

Given the differential influence of dual oxidases on neutrophil migration in the two models and utilizing previous observations that the two models differentially utilize CD18-dependent mechanisms, we hypothesized that the lack of dual oxidases would have no influence on neutrophil marker expression after LPS exposure but would be required for CD18-independent neutrophil activation and migration in the VILI model. As evidence for this differential expression we evaluated neutrophil activation markers CD11a/CD18, CD11b/CD18, L-selectin, and CD49d and compared differential expression in wild-type or *Duoxa* ^*−/−*^ mice exposed to LPS versus VILI.

## Materials and methods

### Definitions

We provided positive-pressure ventilation at a tidal volume of 10 ml/kg with 2 cm H_2_0 positive end-expiratory pressure (PEEP). Recruitment maneuvers (RM) of an inspiratory pressure of 20 cm H_2_0 for 10 s was provided every 20 min. We calculated an SpO_2_/FiO_2_ ratio using 5-min interval readings of SpO_2_ divided by FiO_2_. *Duoxa* ^*−/−*^ (KO) and (*Duox* ^*+/+*^ (WT) mice were divided into groups of non-ventilated KO (NV^−/−^), WT (NV^+/+^), positive-pressure ventilation KO (PPV^−/−^), and positive-pressure WT (PPV^+/+^). Lipopolysaccharide (LPS) groups were exposed to intratracheal administration of LPS for 4 h; KO (LPS IT 4-h^−/−^) and WT (LPS IT 4-h^+/+^).

### Animals

WT mice (129S6/SvEvTac) were obtained from Taconic Biosciences (One Discovery Drive Suite 304; Rensselaer, NY 1244) and were allowed to acclimatize for 1 week prior to study enrollment. KO mice were generated as previously described (Grasberger et al., 2012) and bred in-house on a 129S6 background. All mice were housed in plastic cages over autoclaved bedding in a HEPA-filtered laminar flow cage rack on a 12-h light/dark cycle. Mice were allowed free access to water and a standard diet (Purina Rodent Chow). KO mice are phenotypically hypothyroid, so were initially treated with subcutaneous injections of 40 ng L-T_4_/g body weight from birth to weaning and then supplemented with L-Thyroxine (Sigma) in the drinking water as previously described (Cagle et al., 2021). Procedures with the mice were performed in accordance with an approved IACUC protocol. Mice were routinely monitored and cared for by the veterinary staff at the University of California, Davis in AALAC-accredited facilities.

### Experimental groups and protocol

Mice were assigned to groups consisting of KO (*n* = 5) and WT (*n* = 5) in each group and divided into non-ventilated, positive-pressure ventilation, or LPS IT exposure groups. Positive-pressure ventilation was instituted for 4-h using a FlexiVent (Flexiware 8.1, Scireq, Montreal, QC, Canada). Lipopolysaccharide (*Salmonella enterica* serotype tryphimurium L6143, Millipore Sigma) was administered *via* an intratracheal (IT) route at a dose of 0.1 mg/kg. Mice were humanely euthanized at 4-h post-injection consistent with the UC Davis IAUCAC-approved protocol.

During positive-pressure ventilation, KO and WT mice were sedated with dexmedetomidine (200 mcg/kg, IP), tiletamine-zolazepam (40 mg/kg, IP), and buprenorphine (0.1 mg/kg, IP). Dexmedetomidine (100 mcg/kg, IP), tiletamine-zolazepam (10 mg/kg, IP), and buprenorphine (0.1 mg/kg, SC) were administered at 2 and 3 h after induction throughout the ventilation period to ensure adequate sedation. FiO_2_ was set at 40% for all groups by using an OxyDial to titrate the fractional oxygen delivered (OxyDial, Starr Life Sciences Corp., Oakmont, PA United States). Mice were placed on warming pads and monitored continuously. Heart rate, respiratory rate, SpO_2_ (MouseOx, Starr Life Sciences Corp., Oakmont, PA, United States), and response to noxious stimulation *via* a toe-pinch response were measured every 5 min throughout the ventilatory period. Mice were euthanized at the end of the study period *via* carotid artery exsanguination. Oxygenation status was measured throughout the PPV period by measuring peripheral capillary oxygen saturation (SpO_2_) and calculating the oxygen saturation index (OSI).

### Bronchoalveolar lavage collection and processing

Bronchoalveolar lavage (BAL) was performed using 1 ml aliquots of sterile phosphate-buffered saline. BAL samples were centrifuged at 1,500 rpm for 10 min. BAL supernatant was collected and stored at −80°C for further analysis. BAL cell pellets were resuspended in ammonium-chloride-potassium (ACK) lysing buffer to lyse red blood cells and then resuspended in PBS for white blood cell (WBC) counts. Total live cell counts were obtained using a hemocytometer after staining with trypan blue dye to exclude dead cells. Differential cell counts were obtained *via* manual counts after a cytocentrifuge preparation of the bronchoalveolar lavage fluid stained with Hema3 fixative (Fisher Scientific, Kalamazoo, MI, United States). Differential cell counts involved counting 10 different fields at ×40 with cells classified as macrophage, neutrophil, lymphocyte, eosinophil, or other. Percent differential counts were recorded, and absolute differential counts were calculated. Total protein levels were evaluated in BAL supernatant *via* a standard protein assay measuring albumin levels (Bio-Rad *DC* Protein Assay, Hercules, CA, United States). L-selectin (CD62L) was measured in bronchoalveolar lavage supernatant using a standardized ELISA test kit (Mouse L-Selectin/CD62L, Catalog Number DY576, R&D Systems, Minneapolis, MN, United States).

### Flow cytometry

Bronchoalveolar lavage pellets were stained with antibodies and analyzed on a Beckman Coulter “Cytoflex” 13-color cytometer (Beckman Coulter, Brea, CA, United States). Antibodies used include CD16/32 (TruStain FcX PLUS, Biolegend, Catalog 156603), CD45 (APC-Fire 750, Biolegend, Catalog 147714), Ly-6G (Alexa Fluor 488, Biolegend, Catalog 127626), CD11a/CD18 (PE, Biolegend, Catalog 141006), CD11b/CD18 (APC, Biolegend, Catalog 101212), CD62L (BV421, Biolegend, 104435), CD49d (PE, Biolegend 103608, Catalog 103608), CD44 (AAPC, Biolegend, Catalog 103012), and CD162 (BV421, BD Biosciences, Catalog 562807). We identified live/dead cell ratios by using propidium iodide (PI, Fisher Scientific Catalog BDB556463).

### Statistics

GraphPad Prism 8 (GraphPad Software, Inc.; San Diego, CA, United States) was used for data analysis. Outliers were identified and removed using a robust regression and outlier removal (ROUT) method with coefficient Q set at 1%. Shapiro-Wilk test was used to assess for normality and data was presented in mean ± standard deviation when normally distributed, otherwise reported as median ± interquartile range when not normally distributed. One-way ANOVA with a Tukey correction for multiple comparisons was used to evaluate parametric data and a Kruskal-Wallis test with Dunn’s multiple comparison test was used to evaluate non-parametric data when possible; if not either a Welch’s *t*-test or Mann-Whitney test (two-tailed, unpaired) was used and a Bonferroni correction applied to comparisons with multiple groups. Pearson correlation coefficients were used for data that was normally distributed, whereas a non-parametric Spearman correlation was used for data that was not normally distributed. Flow cytometry data was collected on multiple days throughout the course of the experiment, so normalization of the data was needed. Median fluorescence intensity data was normalized by calculating a gain adjustment value: [(Gain_Sample_–Gain_Initial_)/Gain_Initial_] and then applying the calculated gain adjustment to the MFI as determined during quality control on the day of experimentation to obtain an MFI adjustment value. An adjustment factor was then calculated by taking the MFI from the daily quality control and dividing this by the adjusted MFI value. The adjustment factor was calculated for each fluorescent marker for an individual subject. Outliers were identified by use of a box plot to identify data points outside of the quartile range and further evaluated using a percentage analysis with standard recommended cutoff identified as in the top and bottom 2.5% (two-tailed, *α* = 0.05) or observations above or below ± 2.24 standard deviation units if the data was normally distributed. Grasberger et al. (2012) fold change was calculated by (final value–initial value)/initial value. A predetermined *p*-value of < 0.05 was considered statistically significant, unless otherwise stated.

## Results

**FIGURE 1:**
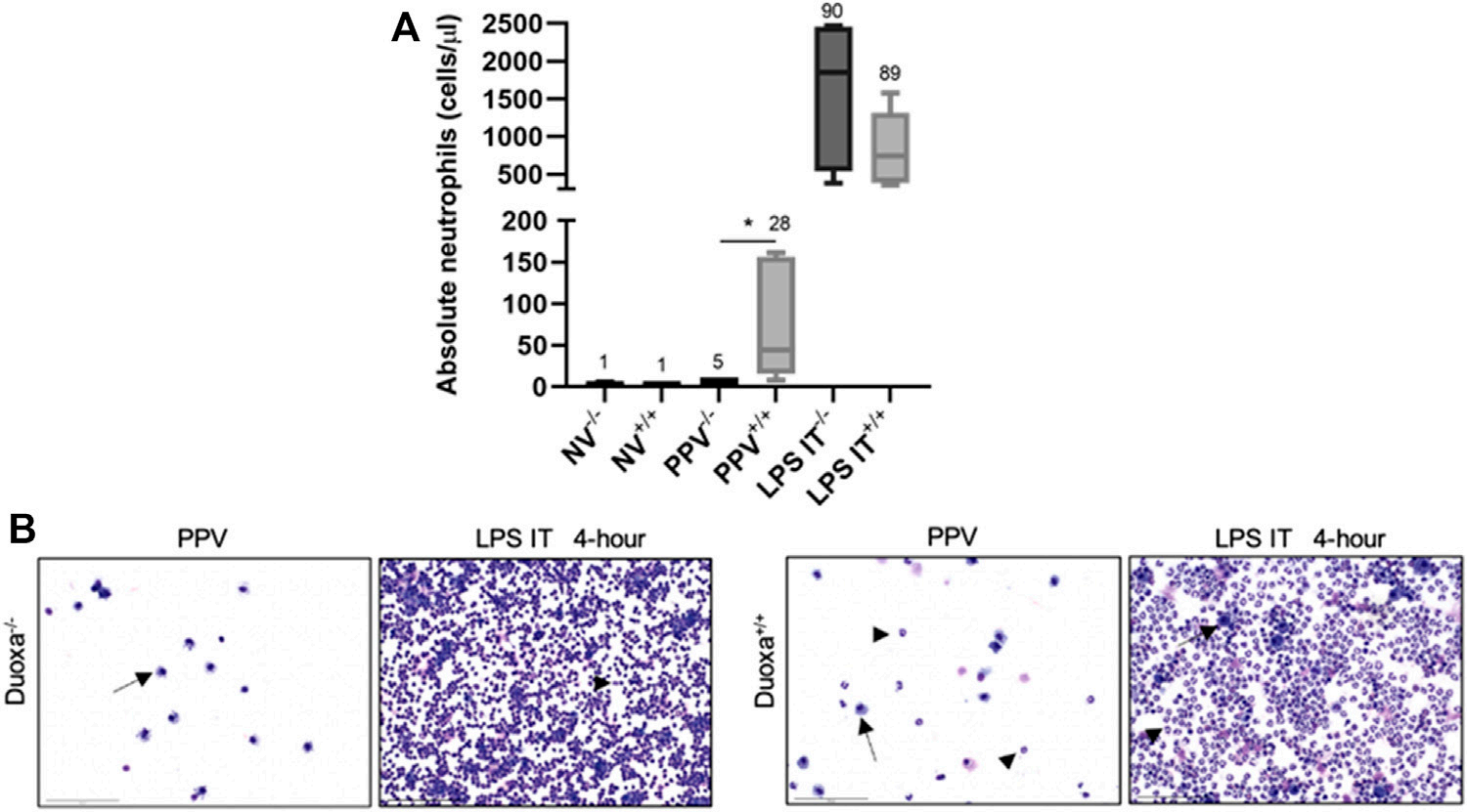
Duox is differentially required for neutrophil migration into the airway based on stimulus. **(A)** Neutrophil counts in lung lavage from Duoxa knockout mice (−/−) and wild-type mice (+/+) that were unventilated (NV), subjected to injurious positive pressure ventilation (PPV), or instilled intratracheally with lipopolysaccharide (LPS). Numbers above each bar represents percent neutrophils compared to total cells counted. *, *p = .004*. **(B)** Bronchoalveolar lavage cytology after positive pressure ventilation (PPV) or LPS instillation in Duoxa knockout (−/−) and wild-type (+/+) mice. Arrows highlight macrophages, arrowheads highlight neutrophils. Images were obtained at ×40 magnification.

We compared the degree of neutrophil recruitment in our VILI and LPS-induced lung injury models. Peripheral blood and bronchoalveolar lavage counts of neutrophils between non-ventilated, ventilated, and LPS-induced injury were measured in *Duoxa* ^*−/−*^ and *Duoxa* ^*+/+*^ mice (Figure 1 and data not shown). As we previously observed (Cagle et al., 2021), there were no significant differences in peripheral blood neutrophils in any of the experimental groups (data not shown). There was a robust increase in airway neutrophilia after intratracheal instillation of LPS in both *Duoxa* ^*−/−*^ and *Duoxa* ^*+/+*^ mice. In contrast, there was increased airway neutrophilia after positive-pressure ventilation only in the *Duoxa* ^*+/+*^ mice (Figure 1).

**FIGURE 2:**
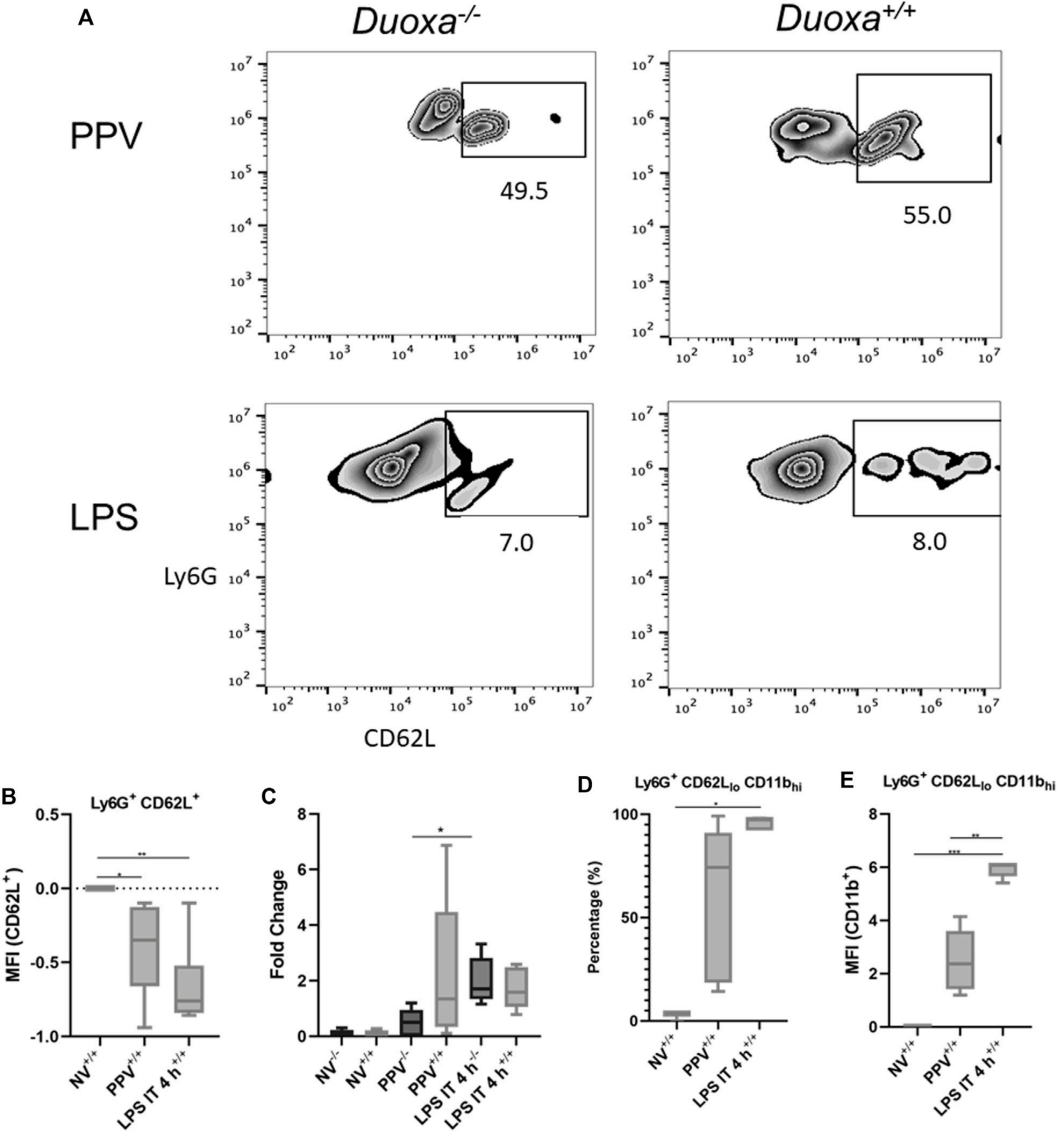
Duox does not impact L-selectin activation or shedding. Duoxa knockout mice (−/−) and wild-type mice (+/+) were subjected to injurious positive pressure ventilation (PPV) or instilled intratracheally with lipopolysaccharide (LPS) followed by isolation of neutrophils from bronchoalveolar lavage (BAL). Neutrophils were isolated using Ly6G antibody and subsequently phenotyped by flow cytometry using CD62L, CD11b antibodies, or both. **(A)** Populations of Ly6G^+^ (*y*-axis), CD62L^+^ (*x*-axis) neutrophils in Duoxa knockout (−/−) compared to wild-type (+/+) mice after exposure to PPV or LPS. **(B)** Mean fluorescence intensity (MFI) of Ly6G^+^, CD62L^+^ neutrophils in PPV- or LPS-exposed mice compared to non-ventilated, untreated controls (*, *p* = .040, **, *p* = .003). **(C)** Protein levels of CD62L by ELISA from BAL fluid in non-ventilated, untreated controls compared to PPV- and LPS-treated mice. Fold-change in protein levels from non-ventilated, untreated controls is shown. Shedding of CD62L ligand was higher in wild-type (+/+) versus Duoxa knockout (−/−) mice after PPV (*, *p* = .010) but not after LPS exposure (*p* = .164). **(D)** Percentage of Ly6G^+^ BAL neutrophils expressing low CD62L and high CD11b levels isolated by flow cytometry after no exposure (NV), PPV, or LPS. (*, *p* = .007 for LPS versus NV). **(E)** MFI of BAL neutrophils gated for CD62L_lo_ and CD11b_hi_ by flow cytometry in non-ventilated, untreated wild-type mice (NV) compared to PPV- and LPS-exposed mice. (**, *p* = .001; ***, *p* = .008).

The fact that the presence or absence of dual oxidase had no effect on neutrophil recruitment in the LPS model but had a significant effect on neutrophil recruitment in our VILI model supported our hypothesis that there are DUOX-dependent and DUOX independent mechanisms of neutrophil recruitment in the lung. We initially looked at differences in L-selectin activation and shedding on neutrophils as an initial potential difference between the various models. Although there was a higher degree of L-selectin positive neutrophils recovered from bronchoalveolar lavage in the VILI model (Ly6^+^/CD62L^+^) compared to the LPS group, there was no significant difference noted between *Duoxa* ^*−/−*^ and *Duoxa* ^*+/+*^ mice in either VILI or LPS models (Figures 2A,B). Similarly, there was no significant difference between all groups when measuring L-selectin protein in bronchoalveolar lavage (Figure 2C). These data suggest dual oxidases do not impact the degree of L-selectin activation or shedding on activated neutrophils in either VILI or after LPS instillation.

Using flow cytometry to determine the degree of activated neutrophils (CD62L_lo_/CD11b_hi_), we observed significant upregulation of activated neutrophils isolated from bronchoalveolar fluid in mice after intratracheal LPS and VILI compared to control mice (Figures 2D,E). Positive pressure ventilation resulted in a lower degree of neutrophil activation compared LPS instillation consistent with the lower overall degree of neutrophil influx seen with VILI compared to LPS.

**FIGURE 3:**
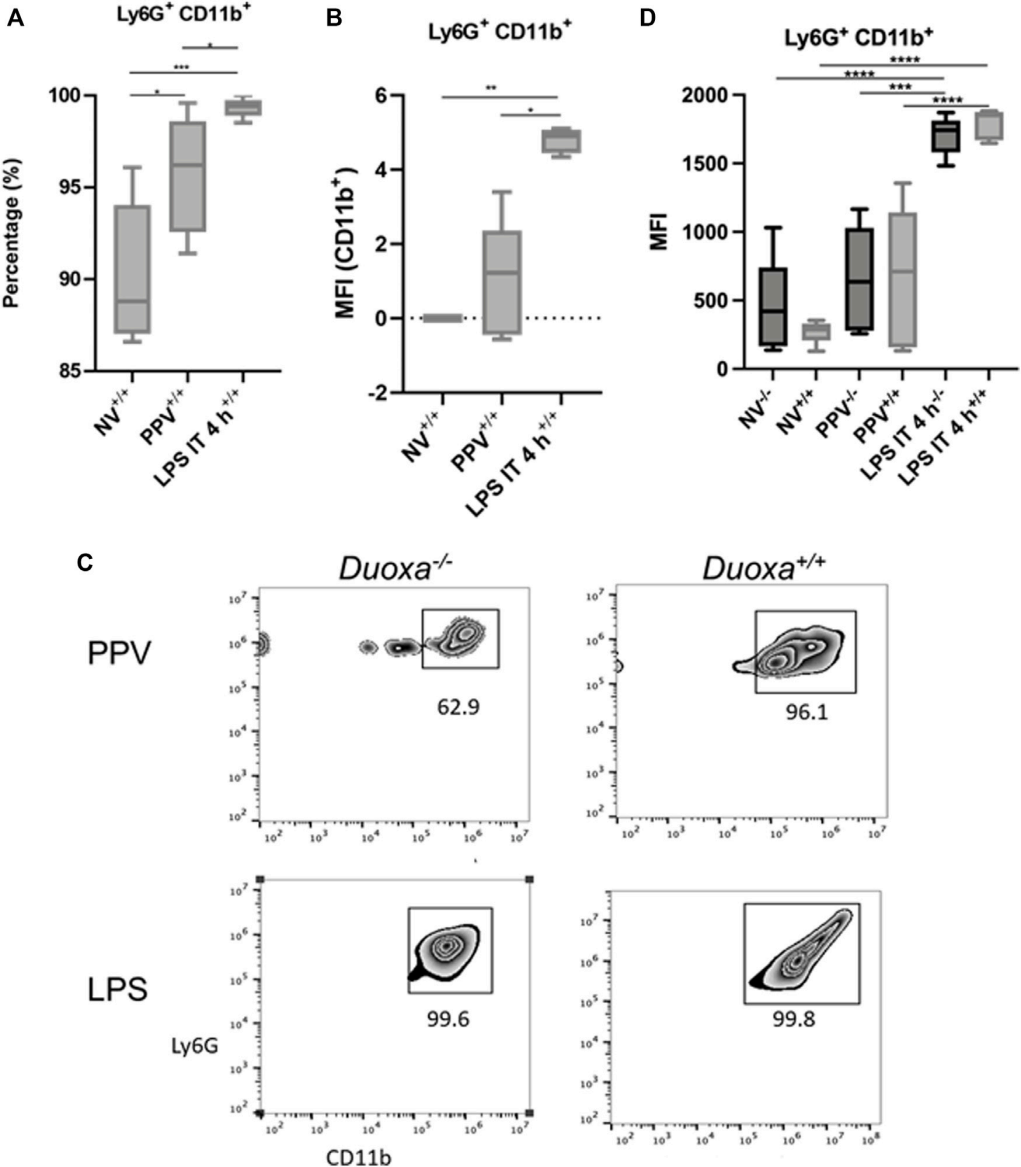
CD18 activation mechanisms are Duox-independent. Duoxa knockout mice (−/−) and wild-type mice (+/+) were subjected to injurious positive pressure ventilation (PPV) or instilled intratracheally with lipopolysaccharide (LPS) followed by isolation of neutrophils from bronchoalveolar lavage (BAL) by flow cytometry using Ly6G antibody and subsequently phenotyped using CD18/CD11b antibodies. **(A)** Percentage of CD11b^+^ neutrophils isolated from BAL fluid in wild-type mice with no exposure (NV), injurious positive pressure ventilation (PPV), or intratracheal LPS (LPS IT) (*, *p* = .029; **, *p* = .0009). **(B)** Mean fluorescence intensity (MFI) of Ly6G^+^, CD18/CD11b^+^ neutrophils in PPV- or LPS-exposed mice compared to non-ventilated, untreated controls (*, *p* = .025, **, *p* = .003). **(C)** Populations of Ly6G^+^ (*y*-axis), CD11b^+^ (*x*-axis) neutrophils in Duoxa knockout (−/−) compared to wild-type (+/+) mice after exposure to PPV or LPS. **(D)** MFI of BAL neutrophils gated for Ly6G^+^ and CD18/CD11b^+^ by flow cytometry isolated from wild-type (+/+) and Duoxa knockout (−/−) mice in non-ventilated, untreated (NV) controls compared to PPV- and LPS-exposed mice. (***, *p* = .002; ****, *p* < .001).

To further evaluate the role of CD18-dependent mechanisms of neutrophil activation, we examined the percentage of neutrophils that were positive for CD11b/CD18 in bronchioalveolar lavage after stimulation. We observed that there was a significantly higher percentage of CD11b-expressing neutrophils after LPS instillation compared to PPV. The degree of CD11b/CD18 expression was slightly higher in the PPV group compared to control, whereas LPS induced a substantial increase in CD11b/CD18 expression on neutrophils (Figures 3A,B). Similarly, dual oxidases appear to not be required for CD11b/CD18 expression after LPS exposure. Nearly 100% of all Ly6G+ neutrophils in the bronchoalveolar lavage fluid express CD11b/CD18 after LPS exposure. In contrast, there was lower expression of CD11b/CD18 in the Duoxa−/− mice, although this was not statistically significant (Figures 3C,D).

**FIGURE 4:**
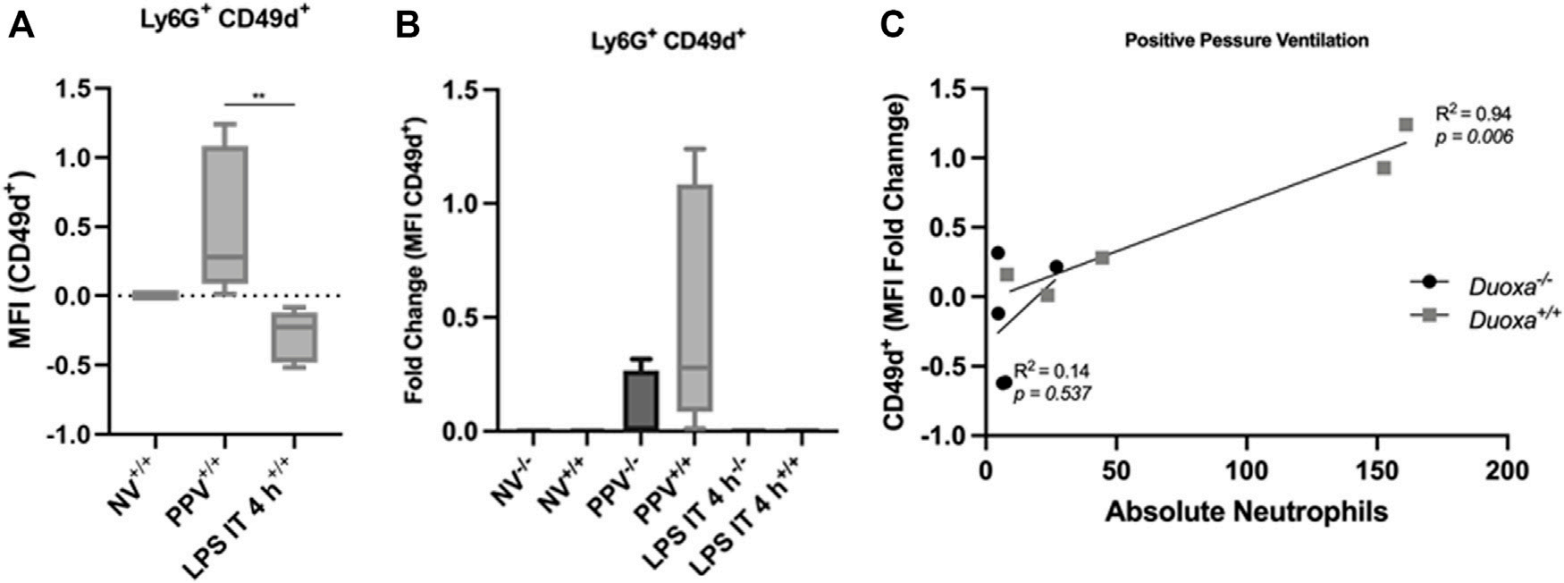
Injurious positive pressure ventilation induces CD18-independent mechanisms that is Duox-dependent. Duoxa knockout mice (−/−) and wild-type mice (+/+) were subjected to injurious positive pressure ventilation (PPV) or instilled intratracheally with lipopolysaccharide (LPS) followed by isolation of neutrophils from bronchoalveolar lavage (BAL) by flow cytometry using Ly6G antibody and subsequently phenotyped using CD49d antibodies. **(A)** Mean fluorescence intensity (MFI) of BAL neutrophils gated for Ly6G^+^ and CD49b by flow cytometry isolated from wild-type (+/+) mice in non-ventilated, untreated (NV) controls compared to PPV- and LPS-exposed mice. (**, *p* = .005). **(B)** Fold change of MFI for Ly6G^+^, CD49d^+^ neutrophils isolated from BAL fluid in non-ventilated, untreated wild-type (+/+) and Duoxa knockout (−/−) mice compared to PPV- and LPS-treated mice. CD49d^+^ expression was higher in wild-type versus knockout mice only in the PPV group (*p = .0079*). **(C)** MFI of Ly6G^+^ CD49d^+^ cells was correlated to absolute neutrophils in bronchoalveolar lavage in mice exposed to PPV. There was a strong correlation seen in wild-type mice (*R* ^2^ = 0.94, *p = .006*) that was not observed in Duoxa knockout mice.

Given our findings that PPV does not appear to stimulate a robust activation of CD18, we compared the differential expression of CD49d between the two models. Consistent with our hypothesis, there was a significant increase in CD49d expression on neutrophils after PPV compared to controls. In contrast, there was a reduction in CD49d expression in mice exposed to LPS compared to control (Figure 4A). In addition, the wild-type mice had a more robust activation of CD49d compared to the knockout mice supporting the role of dual oxidases in the activation of this CD18-independent pathway (Figure 4B). There was a strong correlation between the number of neutrophils in the bronchoalveolar space and the presence of dual oxidase (Figure 4C). Compared to Duoxa+/+ mice, Duoxa−/− mice were not able to demonstrate significant recruitment of neutrophils into the alveolar space after PPV and there was a minimal level of CD49d activation.

## Discussion

We directly compared stretch-induced (VILI) lung injury with LPS-induced lung injury to determine whether dual oxidases play essential roles in both models (Cagle et al., 2021). As a first step, we measured neutrophil activation markers in bronchoalveolar lavage fluid after VILI or IT LPS. Markers of activated neutrophils, namely CD62L_lo_ and CD11b_hi_, were increased in both lung injury groups with LPS-induced injury leading to significantly more activation.

L-selectin (CD62L) and β_2_-integrin (CD11b/CD18) have previously been determined to be essential for pulmonary neutrophil transmigration in lung injury (Doyle et al., 1997; Kubo et al., 1999; Choudhury et al., 2004; Doerschuk 1992; Kuebler et al., 2000; Kuebler et al., 1997), and this was confirmed in our study. Choudhury et al. (2004) identified an association with L-selectin and CD11b/CD18 in neutrophil migration in LPS-induced injury, but only L-selectin was associated with neutrophil migration in VILI, suggesting a CD18-independent pathway (Doerschuk et al., 1992; Oeckler and Hubmayr, 2007; Matthay and Zemans, 2011). CD49d (α4β1 (VLA-4)) was upregulated in the VILI model, but not in the LPS-induced injury groups, suggesting an alternate pathway to neutrophil migration in VILI compared to LPS-induced injury, which is supportive of a CD18-independent pathway in VILI.

Although prior studies established that β_2_-integrins are essential for migration of neutrophils across the airway epithelium after LPS (Choudhury et al., 2004; Doerschuk et al., 1992; Oeckler and Hubmayr, 2007; Matthay and Zemans, 2011), our study adds to this work by identifying CD11b as a key signaling molecule that upregulated in neutrophils in lungs damaged by VILI. It is not surprising that this response is more subtle in VILI-injured mice compared to more overwhelming injury in the IT LPS-induced injury group.

IT LPS-induced injury and VILI resulted in an increasing trend of peripheral blood absolute neutrophil counts compared to non-ventilated controls, indicating an expected increased inflammatory response in all groups. However, the mechanism of neutrophil transmigration into the lung varied between the two models. Evaluation of neutrophil activation markers within bronchoalveolar lavage fluid between VILI and IT LPS- induced lung injury models yielded downregulation of CD62L (L-selectin) in both models with a pronounced upregulation of CD49d in the VILI group compared to the pronounced upregulation of CD11b/CD18 and lack of CD49d expression in the IT LPS-induced injury group.

CD62L is shed upon neutrophil activation and is essential for slowing movement of the neutrophil along the endothelium followed by β2-integrin (CD11a/CD18, CD11b/CD18, CD11c/CD18) activation and firm adhesion to receptors on the endothelium, such as ICAM-1 (CD54) (Simon et al., 1995; Butcher 1991; Springer 1994). CD62L is an integral step in neutrophil transmigration and lack of CD62L shedding can result in decreased neutrophil transmigration. Simon et al. (1995), determined that CD62L (L-selectin) is essential for the initiation of neutrophil rolling along the endothelium, but may also be essential to signal β2-integrin dependent adhesion and migration. Similar expression of CD62L in KO mice compared to wild-type mice indicated a different mechanism affected in neutrophil transmigration between Duoxa^−/−^ and Duoxa^+/+^ mice. We propose based on these observations that dual oxidases are not associated with CD62L shedding from neutrophils during activation, and that there is a mechanism further downstream to explain our observed differences.

Consistent with this observation, Ridger previously determined that CD49d mediates CD18-independent neutrophil accumulation in neutrophil migration in pulmonary inflammation (Ridger et al., 2001). Upregulation of CD49d was noted in the VILI group, but not in the IT LPS-induced injury group, indicating dual oxidases are important for CD18-independent migration. The mechanisms responsible for this induction remains to be determined. Given the very low to absent expression of dual oxidases in immune cells, we postulate that crosstalk between Duox-mediated epithelial signaling and endothelium are responsible for our observations. Previous reports have highlighted the importance of Duox enzymes during tissue damage and the role of Duox-dependent Ca^2+^-mediated signaling in this process (van der Vliet 2014; Guse 2022). Although there is ample *in vitro* literature connecting Duox and TLR4 signaling (Wu et al., 2013; Yamaguchi et al., 2016), our *in vivo* data strongly suggest TLR4/LPS-independent mechanisms during VILI and no role for Duox in LPS-mediated neutrophil sequestration in the lung.

## Conclusion

We hypothesized that there would be fewer activated neutrophils in the lungs of mice with ventilator-induced lung injury and secondarily, less migration of neutrophils into the airway lumen compared to direct LPS-induced lung injury in mice lacking dual oxidase enzymes. Ventilator-induced stretch lung injury and LPS-induced lung injury result in upregulation of CD11b/CD18 and downregulation or shedding of CD62L; although the degree of neutrophil activation is significantly higher in IT LPS exposure compared to our model of VILI. CD49d (α4β1 (VLA-4)) was upregulated in the VILI only, highlighting a potential mechanistic difference for neutrophil migration in VILI injury compared to IT LPS-induced injury. Our data support a CD18-independent pathway in VILI and that this pathway depends on dual oxidases. We suggest that airway epithelium-derived dual oxidases function as a co-factor in neutrophil transmigration *via* upregulation of CD49d (α4β1 (VLA-4)). In addition, the prominent expression of CD49d (α4β1 (VLA-4)) on neutrophils in positive-pressure induced ventilation could serve as a target to limit neutrophil recruitment and subsequent inflammatory injury in positive-pressure ventilation.

## Data Availability

The raw data supporting the conclusion of this article will be made available by the authors, without undue reservation.
