# Supplementary figures and images for: Early mechanisms of neutrophil activation and transmigration in acute lung injury
Source: Front Physiol. 2022 Dec 21;13:1059686. doi: 10.3389/fphys.2022.1059686 (PMC9811384; doi:10.3389/fphys.2022.1059686)

## Slide 1
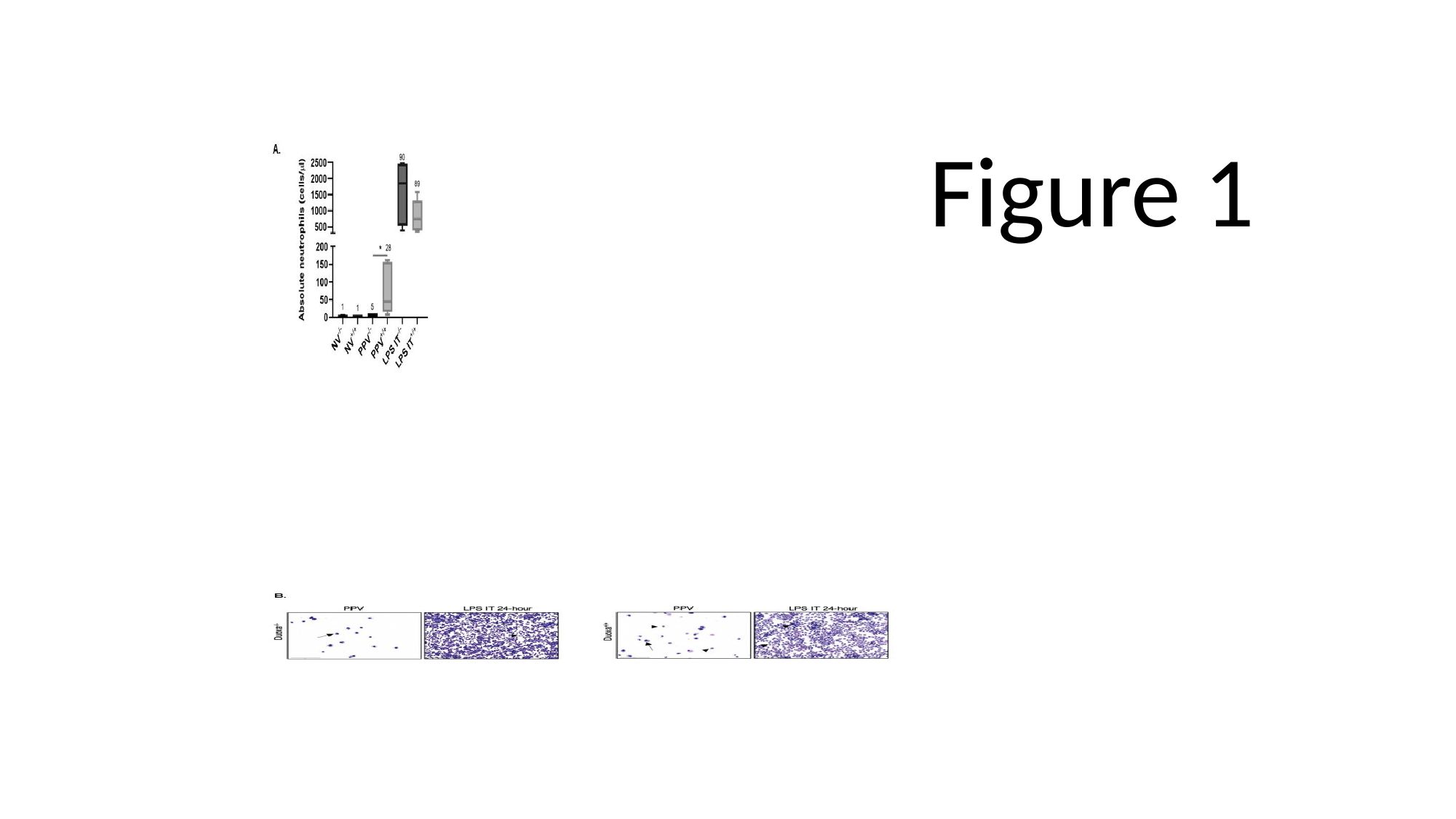

Figure 1

## Slide 2
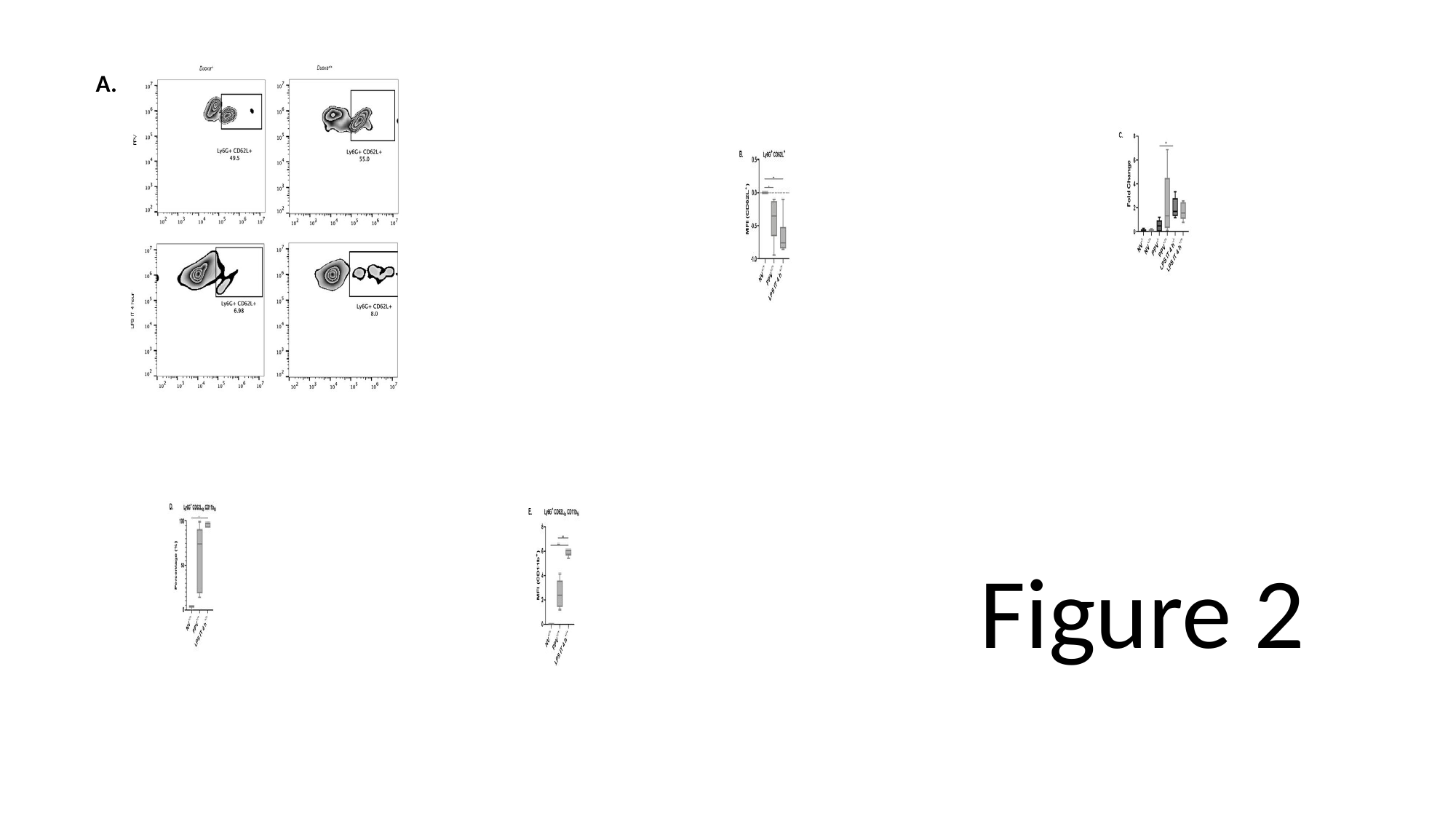

A.
Figure 2

## Slide 3
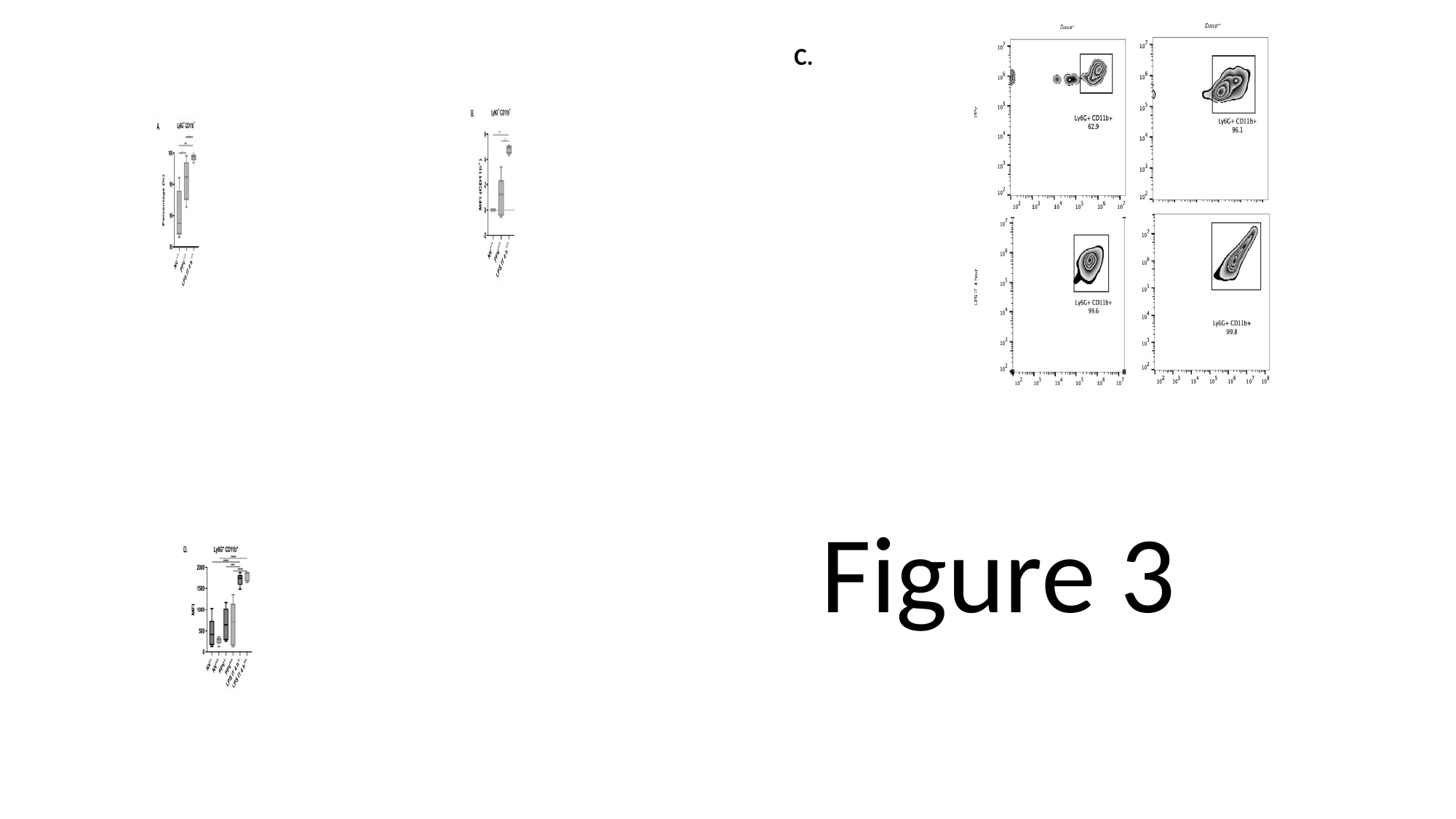

C.
Figure 3

## Slide 4
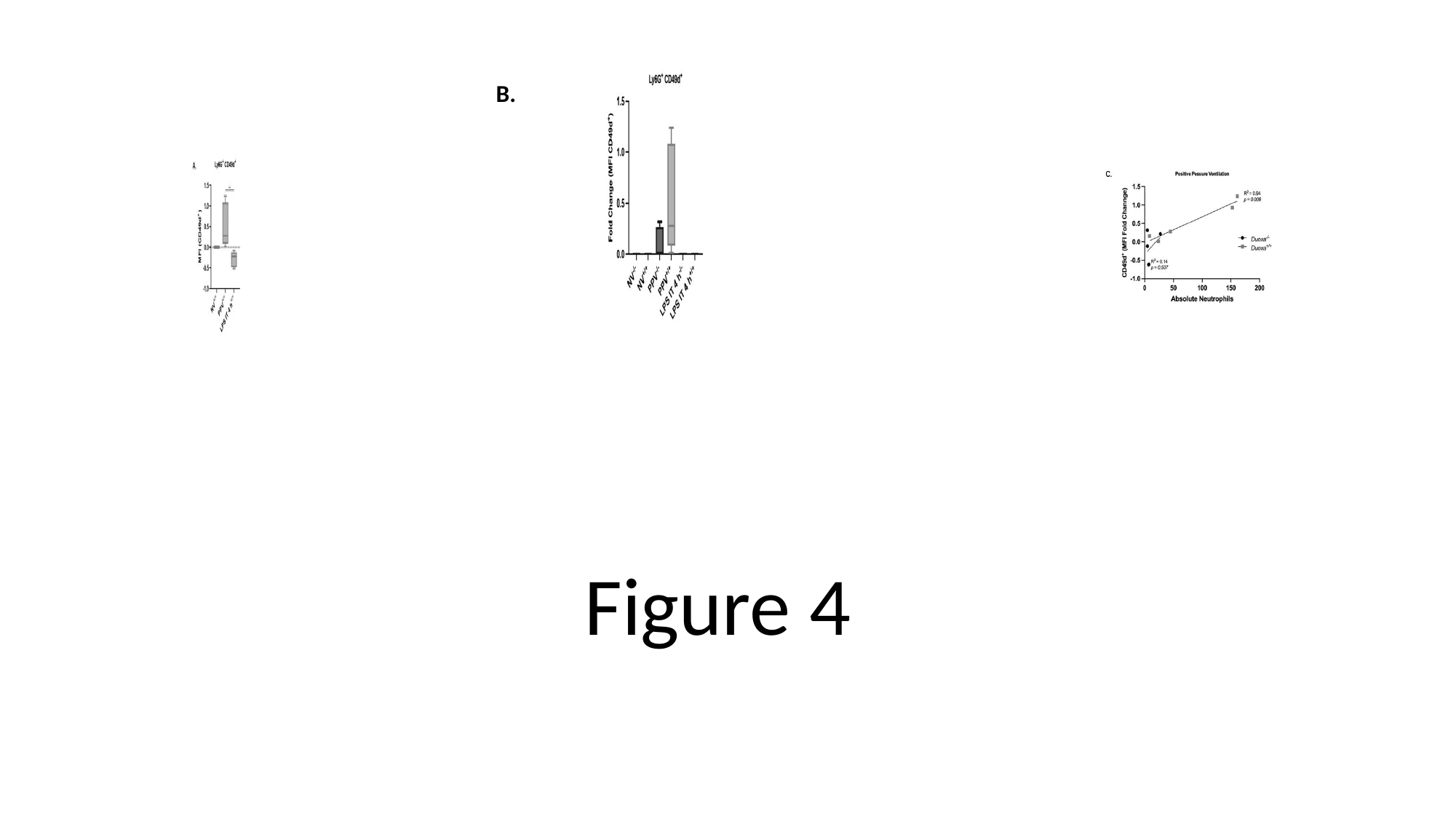

B.
Figure 4

Supplement: Supplementary file 1 [file Presentation1.PPTX]
